# Comprehensive RNA-sequencing analysis of colorectal cancer in a Korean cohort

**DOI:** 10.1016/j.mocell.2024.100033

**Published:** 2024-02-23

**Authors:** Jaeim Lee, Jong-Hwan Kim, Hoang Bao Khanh Chu, Seong-Taek Oh, Sung-Bum Kang, Sejoon Lee, Duck-Woo Kim, Heung-Kwon Oh, Ji-Hwan Park, Jisu Kim, Jisun Kang, Jin-Young Lee, Sheehyun Cho, Hyeran Shim, Hong Seok Lee, Seon-Young Kim, Young-Joon Kim, Jin Ok Yang, Kil-yong Lee

**Affiliations:** 1 Department of Surgery, Uijeongbu St. Mary’s Hospital, College of Medicine, The Catholic University of Korea, Uijeongbu 11765, Republic of Korea; 2 Korea Bioinformation Center (KOBIC), Korea Research Institute of Bioscience and Biotechnology, Daejeon 34141, Republic of Korea; 3 Department of Biochemistry, College of Life Science and Biotechnology, Yonsei University, Seoul 03722, Republic of Korea; 4 Department of Surgery, Seoul National University Bundang Hospital, Seoul National University College of Medicine, Seongnam 13620, Republic of Korea; 5 Precision Medicine Center, Seoul National University Bundang Hospital, Seongnam 13620, Republic of Korea; 6 LepiDyne Co., Ltd., Seoul 04779, Republic of Korea

**Keywords:** Cell cycle, Colorectal neoplasm, DNA replication, RNA, RNA-sequencing

## Abstract

Considering the recent increase in the number of colorectal cancer (CRC) cases in South Korea, we aimed to clarify the molecular characteristics of CRC unique to the Korean population. To gain insights into the complexities of CRC and promote the exchange of critical data, RNA-sequencing analysis was performed to reveal the molecular mechanisms that drive the development and progression of CRC; this analysis is critical for developing effective treatment strategies. We performed RNA-sequencing analysis of CRC and adjacent normal tissue samples from 214 Korean participants (comprising a total of 381 including 169 normal and 212 tumor samples) to investigate differential gene expression between the groups. We identified 19,575 genes expressed in CRC and normal tissues, with 3,830 differentially expressed genes (DEGs) between the groups. Functional annotation analysis revealed that the upregulated DEGs were significantly enriched in pathways related to the cell cycle, DNA replication, and IL-17, whereas the downregulated DEGs were enriched in metabolic pathways. We also analyzed the relationship between clinical information and subtypes using the Consensus Molecular Subtype (CMS) classification. Furthermore, we compared groups clustered within our dataset to CMS groups and performed additional analysis of the methylation data between DEGs and CMS groups to provide comprehensive biological insights from various perspectives. Our study provides valuable insights into the molecular mechanisms underlying CRC in Korean patients and serves as a platform for identifying potential target genes for this disease. The raw data and processed results have been deposited in a public repository for further analysis and exploration.

## INTRODUCTION

The conventional therapeutic approach for colorectal cancer (CRC) involves the administration of chemotherapy after surgery, depending on the tumor status, when curative surgery remains a viable option. Despite the implementation of these treatments, mitigating the risk of CRC recurrence remains a challenge.

CRC management primarily relies on the American Joint Committee on Cancer staging system because of its well-established association with oncological prognosis. This staging system traditionally indicates a poorer prognosis with advancing stages; that is, certain subsets of patients with higher disease stages exhibit worse outcomes than their counterparts with lower disease stages (Chu et al., 2016). In this milieu, a molecular approach is imperative because of a lack of clinical evidence that differentiates the survival rates among patients with the same American Joint Committee on Cancer stage.

Recent advancements in high-throughput RNA-sequencing (RNA-Seq) technology have provided a comprehensive platform for exploring the transcriptomic landscape of CRC, enabling the identification of dysregulated genes, signaling pathways, and potential therapeutic targets (The Cancer Genome Atlas Network, 2012). The introduction of the Consensus Molecular Subtype (CMS) classification in 2015 has provided a novel perspective for understanding cancer characteristics, transcending the traditional staging paradigm (Guinney et al., 2015).

In this study, we aimed to use RNA-Seq data to conduct a comprehensive comparative analysis of cancerous and the adjacent normal tissues from a Korean cohort. Furthermore, we endeavored to elucidate the distinct oncological prognostic implications associated with the CMS classification, thereby contributing to a more nuanced understanding of CRC outcomes.

## MATERIALS AND METHODS

### Participant Recruitment

Patients with histologically confirmed colon or rectal cancer (stages II-IV), who were eligible for surgery to achieve tumor resection and obtain adjacent normal tissue in the primary or metastatic sites, were recruited from Uijeongbu St. Mary’s Hospital and Bundang Seoul National University Hospital. Patients who did not consent to participate in the study or to the use their tissues for research were excluded from the study. The datasets used in the present study comprised 214 Korean participants (comprising a total of 381 samples—169 normal and 212 tumor samples) from the Seoul National University Bundang Hospital (BSN; 219 samples) and The Catholic University Uijeongbu St. Mary’s Hospital (CMC; 162 samples). Among them, 212 tumor samples were from BSN (129) and CMC (83), and 169 adjacent normal samples were from BSN (90) and CMC (79).

### Ethics Statement

This study was conducted in accordance with the principles outlined in the Declaration of Helsinki and was approved by the Institutional Review Board (IRB) of St. Mary's Hospital at Uijeongbu (IRB approval number: XC17TNDI0068), Bundang Seoul National University Hospital (IRB approval number: B-1709-423-306), and Yonsei University (IRB approval number: 7001988-201910-BR-727-02). Informed consent was obtained from all participants prior to their inclusion in the study.

Participants were provided detailed information about the study objectives, procedures, potential risks, and benefits. They were given ample time to ask questions and clarify any doubts they may have had. Written informed consent was obtained from each participant, and a copy of the signed consent form was provided to them for their records.

### RNA Extraction and Sequencing

The tumor and adjacent normal fresh-frozen tissues obtained from patients with CRC were dissected into 20 to 40-mg pieces and homogenized 3 to 4 times for 15 seconds at a frequency of 30 Hz using a Tissue Lyser II (Qiagen). RNA was extracted using the RNeasy Mini Kit (Qiagen) following a standard animal tissue RNA extraction protocol. Total RNA concentration was determined using Quant-IT RiboGreen (R11490; Invitrogen). To assess RNA integrity, the samples were run on the TapeStation RNA ScreenTape system (5067-5576; Agilent). Only high-quality RNA preparations (RNA integrity number >7.0) were used for RNA library construction. Thereafter, the total RNA was subjected to rRNA degradation using the Ribo-Zero Gold rRNA Removal Kit (MRZG12324; Illumina) and then mixed with 2 μL of 100-fold diluted ERCC Mix2 solution from the ERCC RNA Spike-In Mix Kit (4456740; Ambion). An RNA-Seq library was prepared using the TruSeq RNA Sample Prep Kit (Illumina). The libraries were subsequently subjected to paired-end (read length 2 × 100 bp) sequencing on an Illumina HiSeq2000 platform (Illumina) at the Macrogen Inc. (Gangnam-gu).

### RNA-Seq Analysis

The quality of the FASTQ files was analyzed using multiQC (v.1.15) (Ewels et al., 2016). The GRCh38 reference genome and the gene annotation GTF file (GENCODEv27) of humans were downloaded from GENCODE (https://www.gencodegenes.org/human/). ERCC sequences and FASTA and GTF annotations were downloaded from Thermo Fisher (https://assets.thermofisher.com/TFS-Assets/LSG/manuals/ERCC92.zip), and genome indexing was performed using STAR (v.2.7.8a) (Dobin et al., 2013). The sequenced reads were mapped to the human genome (hg38) using STAR (v.2.7.8a), and gene expression levels were quantified using the count module in RNA-Seq by Expectation-Maximization (RSEM) v.1.3.3 (Li and Dewey, 2011). The edgeR package (v.3.32.2) (Robinson et al., 2010) was employed for batch effect correction using counts (RSEM) in accordance with the "Normalization" section. For the normalization of count data, we utilized the trimmed mean of M-values and applied a model-based quantile-adjusted conditional maximum likelihood method for batch effect removal. Differentially expressed genes (DEGs) were selected from the normalized count data between tumor and normal samples (fold change > 2, false discovery rate < 0.05) following the “The classic edgeR pipeline” section in edgeR. Meanwhile, the trimmed mean of M-values normalized counts per million (CPM) value of each gene was added to 1 and log_2_-transformed for further analysis. A heatmap was generated using the R (v.3.5.0, https://www.r-project.org/) complex heatmap package (v.2.16.1) (Gu et al., 2016). The multidimensional scaling (MDS) plot was generated using the plotMDS function in edgeR, using normalized gene values obtained above in the analysis. CMS classification analysis was performed using CMScaller (Eide et al., 2017) based on RNA-Seq data.

### Filtering of RNA-Seq Data

The RNA-Seq data were subjected to the following steps of filtering during the analysis. From the entire set of samples (comprising a total of 381 samples including normal and 212 tumor samples), we excluded 7 samples with low-quality mapping reads (*N* = 374). Additionally, k-means clustering (k = 2) identified and excluded 32 samples that exhibited mixing between normal and tumor tissues (*N* = 342). As a part of data preprocessing, RSEM and normalized read counts for each sample were recalculated, and log2CPM values were compiled. The processed data have been uploaded to Zenodo (see Data Availability Statement).

### Gene Set Enrichment Analysis

Gene set enrichment analysis (GSEA) was performed to compare tumor and normal tissues using the GSEApy tool (v. 0.10.1, https://github.com/zqfang/GSEApy) (Subramanian et al., 2005) with categories of Kyoto Encyclopedia of Genes and Genomes pathways, biological processes, WikiPathways, Jensen DISEASES (Disease-gene associations mined from literature), Disease Perturbations from Gene Expression Omnibus (GEO), and RNA-Seq GEO Signatures in the enrichR library (https://maayanlab.cloud/Enrichr/#libraries) (Kuleshov et al., 2016). For GSEA, a significance threshold of *P* < .05 was employed to identify significantly enriched gene sets. At the sample level, a positive correlation was defined for tumor samples, whereas a negative correlation was specified for normal samples. Figures 2D and S2C were generated using the gseaplot function from GSEApy with gene sets obtained from the “Disease Perturbations from GEO up” GMT file, specifically including “colorectal adenocarcinoma DOID-0050861 human GSE24514 sample 595,” “colorectal cancer DOID-9256 human GSE32323 sample 552,” “colon adenoma DOID-0050912 human GSE4183 sample 550,” and “colon carcinoma DOID-1520 mouse GSE422 sample 651.”

### Statistical Analyses

To assess the significance of clinical information between the Tumor-weak (*N* = 30) and the remaining tumor samples (*N* = 155), statistical analyses were conducted using the fisher_test function in the stats package (v.3.6.2, https://stat.ethz.ch/R-manual/R-devel/library/stats/html/00Index.html) in R.

Survival analysis was conducted using the ggforest and ggsurve modules in Survminer (v.0.4.9; https://github.com/kassambara/survminer). Among the clinical variables, age was dichotomized into 2 groups—old and young—based on the median value derived from the analysis.

### Subtyping CMCBSN Tumor Samples Using CMScaller and Non-negative Matrix Factorization Analysis

Using the RNA-Seq data of the tumor samples (*N* = 187) of the CMCBSN patient group, subtype classification based on the CMS was performed using the standard workflow of CMScaller (Eide et al., 2017). Quantile normalization and log2 transformation were performed on RSEM values as input using the RNAseq = TRUE parameter of the CMScaller module. The CMS template genes (*N* = 458) were derived from the templates from the CMS file of the CMScaller module, and a heatmap was generated by representing log_2_CPM values for each template gene after z-normalization.

For tumor samples (*N* = 187) from the CMCBSN patient group, RNA-Seq data were employed in the estimation of factorization rank using non-negative matrix factorization (NMF) analysis. The analysis was conducted using the standard workflow of the NMF R package (v.0.26) (Gaujoux and Seoighe, 2010). Using the nmf module, the log_2_CPM values were set as input, and analyses were conducted from ranks 2 to 7, with 500 iterations. The analyses were performed separately on genes (*N* = 19,550) after the normalization process mentioned above, genes with a standard deviation of 0.5 or higher (*N* = 8,886), and genes designated as CMS template genes (*N* = 458). For the estimation of the factorization rank, the value of “r” was determined as the first point at which the cophenetic coefficient began to decrease.

### Comparative Analysis Among DNA Methylome, DEGs, and CMS Subtypes

The DNA methylation data (Infinium Methylation EPIC array; EPIC array) utilized in this study were derived from previously published data for the same cohort (Lee et al., 2023). This analysis involved preprocessing with the minfi (v.1.48.0) (Aryee et al., 2014) R package, utilizing the preprocessSWAN and Combat modules to perform probe type normalization and batch correction from IDAT files. Low-quality probes with a detection *P*-value of <.05 were removed. Based on EPIC array annotation, promoters were classified into TSS200, TSS1500, 1st exon, 5′UTR, and gene body, whereas gene body regions included gene body, 3′UTR, and ExonBnd.

An analysis was conducted on samples with overlapping RNA-Seq data (57 normal and 167 tumor samples), and differentially methylated probes were categorized based on DEGs, using the following criteria: fold change >10% and q-value <0.05. Similarly, in the CMS subtype analysis, DMMPs were identified by comparing each CMS type with the remaining group (fold change > 10% and q-value < 0.05) for CMS template genes.

## RESULTS

### Characteristics of the Study Cohort and RNA-Seq Preprocessing

**Fig. 1:**
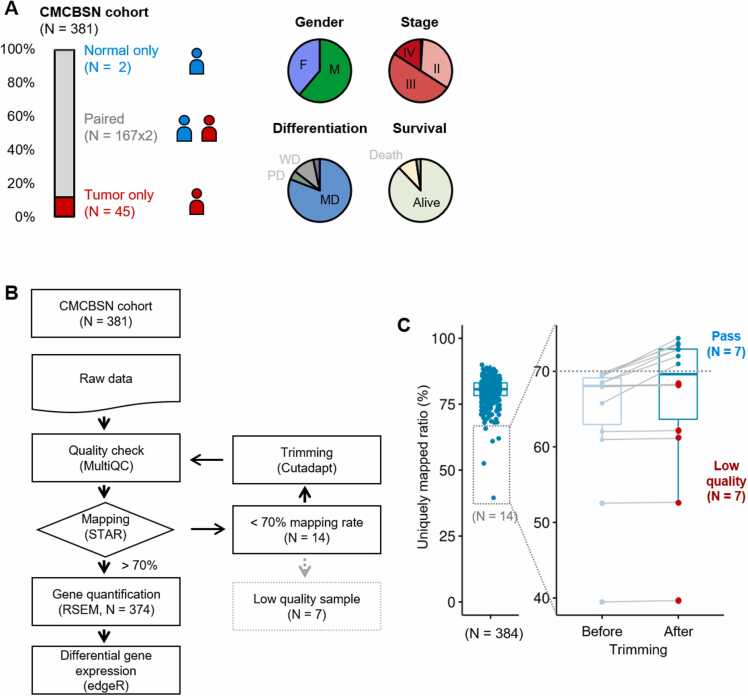
Characteristics of the St. Mary's Hospital and Bundang Seoul National University Hospital (CMCBSN) cohort and RNA-sequencing preprocessing. (A) Clinical information of the 214 Korean participants (comprising a total of 381 samples—169 normal and 212 tumor samples) from the CMCBSN cohort. Distribution of matched tumor (*N* = 212) and normal samples (*N* = 169) (*left*). Distribution of clinical information for the cohort (*right*). (B) Pipeline used for RNA-sequencing data analysis in this study. (C) Preprocessing steps excluding low-quality samples.

To obtain the transcriptomic profile, we generated RNA-Seq data from a cohort of 214 Korean participants (comprising a total of 381 samples, including 169 normal and 212 tumor samples) with CRC recruited from Uijeongbu St. Mary’s Hospital and Bundang Seoul National University Hospital (CMC + BSN; CMCBSN). The cohort had a relatively higher proportion of samples that matched between tumor and normal tissues than those that were unmatched (Fig. 1A, Table S1). The characteristics of the study cohort are presented in Table 1. The average age of the patients was 66 years, with male dominance (*N* = 131, 61.2%). The proportion of patients with pathological stages 1, 2, 3, and 4 was 1.9%, 38.9%, 43.0%, and 16.4%, respectively. Most patients had microsatellite stability (84.6%). The degree of tumor differentiation was moderate in most patients (80.8%). Quality control measures were conducted on the generated RNA-Seq data (Fig. S1A, Table S2). The FASTQ files were aligned using STAR, and read counts were measured using RSEM (Fig. 1B) (Li and Dewey, 2011, Dobin et al., 2013). Based on a report that quality-based trimming can strongly influence the estimation of gene and isoform expression levels (Williams et al., 2016), we performed the analysis without trimming.

**Table 1:** Characteristics of the St. Mary’s Hospital and Bundang Seoul National University Hospital cohort

	Value
Age (years)	66 (IQR 57–75)
Sex	
Male	131 (61.2%)
Female	83 (38.8%)
T	
2	9 (4.2%)
3	162 (75.7%)
4a	36 (16.8%)
4b	7 (3.3%)
N	
0	90 (42.1%)
1	66 (30.8%)
2	58 (27.1%)
M	
0	180 (84.1%)
1	34 (15.9%)
Stage	
1	4 (1.9%)
2	83 (38.8%)
3	92 (43.0%)
4	35 (16.4%)
MSI	
MSS	181 (84.6%)
MSI-L	17 (7.9%)
MSI-H	16 (7.5%)
K-Ras	
Wild	171 (79.9%)
Mutant	43 (20.1%)
Differentiation	
WD	23 (10.7%)
MD	173 (80.8%)
PD	9 (4.2%)
Mucinous	5 (2.3%)
Location	
Right	52 (24.3%)
Left	109 (50.9%)
Rectum	51 (23.8%)
Synchronous	2 (0.9%)

MSI, microsatellite instability; MSI-H, high degree of microsatellite instability.

Subsequently, we performed trimming only for samples with a mapping rate of <70% and excluded samples that still retained a mapping rate of <70% (Fig. 1C). Although the uniquely mapped reads from the RNA-Seq data produced by the 2 hospitals (CMC and BSN) showed different patterns, we considered all of them to be appropriate as they mapped to >20 million reads and were not filtered based on quantity (Fig. S1B, Table S2).

We preprocessed the RNA-Seq data from our cohort of 214 Korean participants with CRC from CMCBSN and performed quality control measures on both qualitative and quantitative aspects, resulting in the exclusion of 7 samples.

### Analysis of DEGs Between Normal and CRC Tissues in the CMCBSN Cohort

**Fig. 2:**
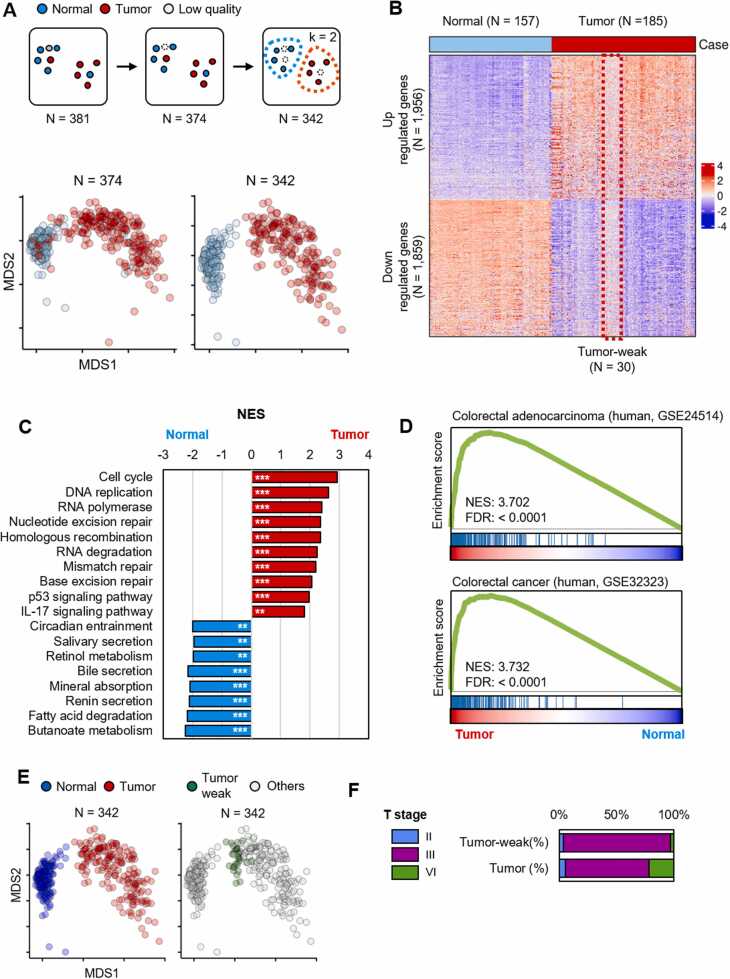
Differentially expressed gene (DEG) analysis between tumor and normal tissues in the St. Mary's Hospital and Bundang Seoul National University Hospital cohort. (A) Filtering process using k-means clustering (k = 2) in RNA-sequencing analysis to separate mixed tumor and normal samples (*top*). Comparison of samples before (*left*) and after (*right*) filtering using a multidimensional scaling (MDS) plot (*bottom*). (B) Heatmap of DEGs between tumor and normal tissues. (C, D) Comparative analysis of tumor and normal tissues using gene set enrichment analysis. (C) Kyoto Encyclopedia of Genes and Genomes pathway enrichment analysis. *P*-values are denoted as follows: *<.05, **<.01, and ***<0.001. (D) Gene Expression Omnibus signatures of RNA-sequencing data. (E) MDS plot comparing normal vs tumor samples (*left*) and the Tumor-weak (*N* = 30) vs remaining tumor groups (*N* = 155) (*right*). (F) Frequency distribution of T stage between the Tumor-weak and remaining tumor groups.

To accurately analyze DEGs between the normal and CRC tissues, we excluded certain samples (*N* = 32) that exhibited mixed patterns between tumor and normal samples according to the k-means clustering (k = 2) results (Figs. 2A and S2A). We then recalculated the DEGs between normal and tumor tissues (Fig. 2B, Table S3). The RNA-Seq data did not show a batch effect between the 2 institutions (CMC and BSN; Fig. S2B). We conducted GSEA using gene sets, including Kyoto Encyclopedia of Genes and Genomes pathways, biological processes, and GEO signatures, to compare the CRC and normal samples using enrichR (Table S4) (Kuleshov et al., 2016). The upregulated genes in tumor tissues were associated with cell cycle, DNA replication, mismatch repair, and IL-17, whereas the downregulated genes were related to metabolism (Fig. 2C). IL-17 promotes CRC development and can be used as a diagnostic marker of CRC (Razi et al., 2019). Additionally, a comparison was conducted targeting gene sets upregulated in CRC compared with those in normal tissues in the GEO dataset (Figs. 2D and S2C). The analysis showed a significant similarity between the upregulated genes in our tumor samples and those in the reference dataset of tumor versus normal conditions. The details of these genes are summarized in Table S4. In conclusion, the DEGs found between the normal and tumor tissues in our RNA-Seq data showed transcriptomic characteristics similar to those of other CRCs. Even after filtering out samples that showed mixing between tumor and normal tissues, some samples still showed a weak pattern in terms of DEGs between tumor and normal samples, and they were termed Tumor-weak samples (Fig. 2B). To precisely identify Tumor-weak samples, we utilized k-means clustering (k = 10) to identify samples (*N* = 30) displaying a weak pattern (Fig. S2D). These samples were relatively closer to normal tissues than to tumor tissues (Fig. 2E). A comparison of clinical information between the Tumor-weak and remaining tumor groups (*N* = 155) revealed significant differences only in the T stage, with a relatively higher proportion of stage VI samples in the remaining tumor group (Figs. 2F and S2E, and Table S5). Considering the possibility of close relationships between the matched normal and tumor samples, statistical analysis was conducted, but no significant findings were obtained (Fig. S2E). In summary, the Tumor-weak samples exhibited a relatively normal tissue-like profile and showed no significant association with those paired with normal samples.

### CMS Classification of RNA-Seq Data From the CMCBSN Cohort

**Fig. 3:**
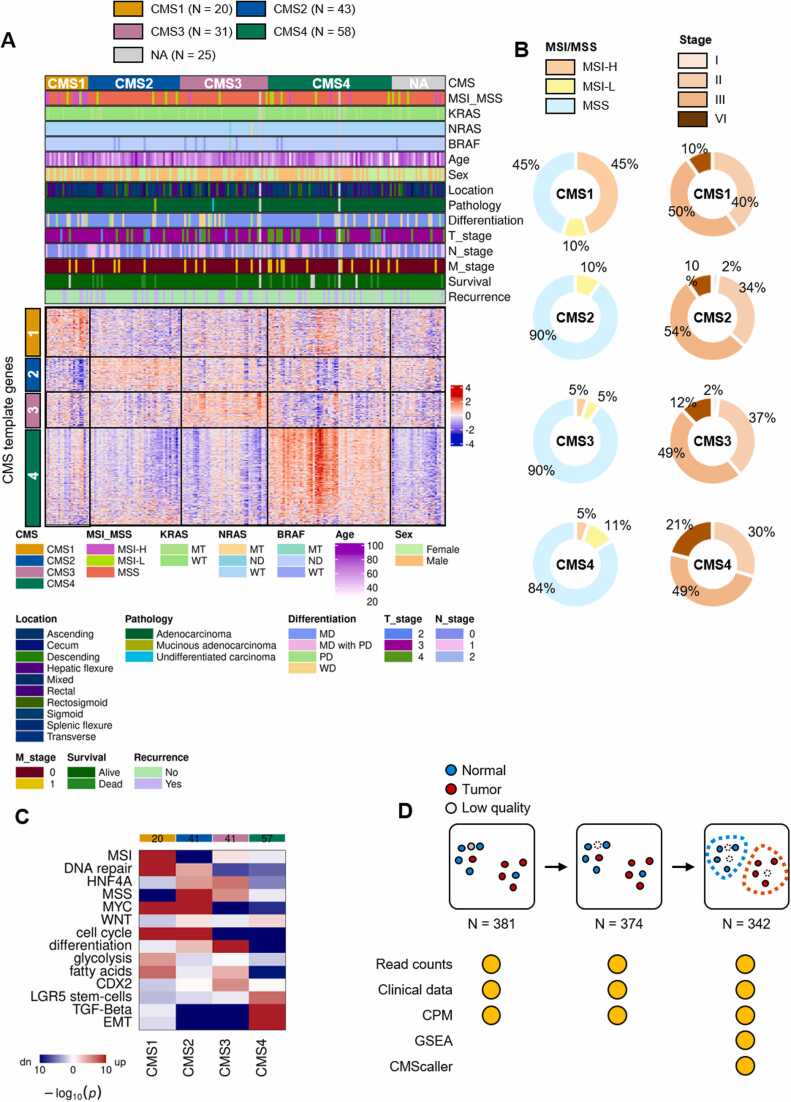
Consensus Molecular Subtype (CMS) classification of the St. Mary's Hospital and Bundang Seoul National University Hospital (CMCBSN) cohort. (A) CMS classification was performed using only tumor samples (*N* = 187) in the CMCBSN cohort and is presented as a heatmap with other clinical data. White color represents NA values. (B) Distribution of microsatellite instability (MSI) types by CMS group (*left*) and stage distribution (*right*). (C) Gene set enrichment analysis comparing the CMS groups. (D) The data provided vary depending on the filtering steps.

Patients with CRC exhibit diverse outcomes and responses to drugs, underscoring the necessity for a comprehensive understanding. The classification of patients with CRC into CMS subtypes, identified through an international collaboration (CMS1, CMS2, CMS3, and CMS4), is a robust and biologically interpretable classification system (Guinney et al., 2015). This framework provides a foundation for precise clinical stratification and targeted interventions in CRC research. We conducted a CMS subtype analysis of our CMCBSN cohort to contribute to this understanding. The CMS classification analysis was conducted exclusively on patients with cancer from the CMCBSN cohort (*N* = 187) based on RNA-Seq data using CMScaller (Eide et al., 2017). The identified subtypes were then summarized using a heatmap, which was complemented with clinical data (Fig. 3A and Table 2). Consistent with the findings of a previous study, a high degree of microsatellite instability was most prevalent in CMS1, and T4 was most common in CMS4, which is known to be associated with worse overall survival (Guinney et al., 2015) (Fig. 3B). Significant verification of a higher prevalence of CMS1 in MSI as indicated by the bold value in Table 2. The CMS1 and CMS4 subtypes have been previously associated with a less favorable prognosis than other CMS categories (Guinney et al., 2015). However, in the survival analysis based on CMS classification, no significant differences were observed among the CMS groups (Fig. S3A and B). These findings could be attributed to the absence of reported patient mortality within the CMS1 subgroup of the CMCBSN cohort, whereas a relatively higher incidence of mortality was observed in the CMS4 subgroup (Fig. S3C, Table S6). Additional analysis of DEGs was performed by comparing CMS subtypes with the remaining groups (Fig. S3D, Table S6). Furthermore, hazard ratios were computed for each clinical dataset, including CMS subtypes (Fig. S4A).

**Table 2:** Analysis of the relationship between Consensus Molecular Subtype (CMS) types and clinical information from the St. Mary's Hospital and Bundang Seoul National University Hospital cohort

	CMS 1	CMS 2	CMS 3	CMS 4	*P*-value (CMS 1 vs others)	*P*-value (CMS 2 vs others)	*P*-value (CMS 3 vs others)	*P*-value (CMS 4 vs others)
Age (years)	64.0 ± 15.1	65.7 ± 11.8	65.3 ± 8.7	63.4 ± 13.4	.678	.829	.961	.658
Sex					.805	.25	>.999	.216
Male	13 (65.0%)	30 (69.8%)	25 (62.5%)	32 (56.1%)				
Female	7 (35.0%)	13 (30.2%)	15 (37.5%)	25 (43.9%)				
T					.136	.076	.814	**.014**
2	1 (5.0%)	3 (7.0%)	2 (5.0%)	3 (5.3%)				
3	18 (90.0%)	35 (81.4%)	29 (72.5%)	37 (64.9%)				
4a	1 (5.0%)	5 (11.6%)	8 (20.0%)	12 (21.1%)				
4b	0	0	1 (2.5%)	5 (8.8%)				
N					.159	.293	.069	.884
0	13 (65.0%)	20 (46.5%)	12 (30.0%)	24 (42.1%)				
1	2 (10.0%)	15 (34.9%)	14 (35.0%)	16 (28.1%)				
2	5 (25.0%)	8 (18.6%)	14 (35.0%)	17 (29.8%)				
M					>.999	.322	.791	.13
0	18 (90.0%)	39 (90.7%)	35 (87.5%)	46 (80.7%)				
1	2 (10.0%)	4 (9.3%)	5 (12.5%)	11 (19.3%)				
Stage					.117	.247	.297	.226
1	0	2 (4.7%)	1 (2.5%)	1 (1.8%)				
2	13 (65.0%)	18 (41.9%)	11 (27.5%)	22 (38.6%)				
3	5 (25.0%)	19 (44.2%)	23 (57.5%)	22 (38.6%)				
4	2 (10.0%)	4 (9.3%)	5 (12.5%)	12 (21.1%)				
MSI					**<.001**	.05	.076	.419
MSS	9 (45.0%)	39 (90.7%)	37 (92.5%)	49 (86.0%)				
MSI-L	2 (7.2%)	4 (9.3%)	2 (5.0%)	5 (8.8%)				
MSI-H	9 (45.0%)	0	1 (2.5%)	3 (5.3%)				
K-Ras					.369	.763	.729	.414
Wild	18 (90.0%)	34 (79.1%)	33 (82.5%)	44 (77.2%)				
Mutant	2 (10.0%)	9 (20.9%)	7 (17.5%)	13 (22.8%)				
Differentiation					.486	.974	.267	.642
WD	3 (15.0%)	4 (9.3%)	7 (18.9%)	5 (8.8%)				
MD	14 (70.0%)	37 (86.0%)	27 (73.0%)	49 (86.0%)				
PD	2 (10.0%)	1 (2.3%)	3 (8.1%)	1 (1.8%)				
Mucinous	1 (5.0%)	1 (2.3%)	0	2 (3.5%)				
Location					.277	.55	.602	.185
Right	8 (40.0%)	9 (20.9%)	10 (25.0%)	14 (24.6%)				
Left	6 (30.0%)	25 (58.1%)	18 (45.0%)	29 (50.9%)				
Rectum	6 (30.0%)	9 (20.9%)	12 (30.0%)	13 (22.8%)				
Synchronous	0	0	0	1 (1.8%)				

MSI, microsatellite instability; MSI-H, high degree of microsatellite instability.

In summary, by analyzing the association between CMS and clinical information, we confirmed the similarity with previous reports (Guinney et al., 2015), thereby verifying the consistency of our data. We also provide a summary of the data at each stage of the filtering process, as shown in Figure 3D.

### Comparing CMS Classification and NMF-based Classification of RNA-Seq Data From the CMCBSN Cohort

**Fig. 4:**
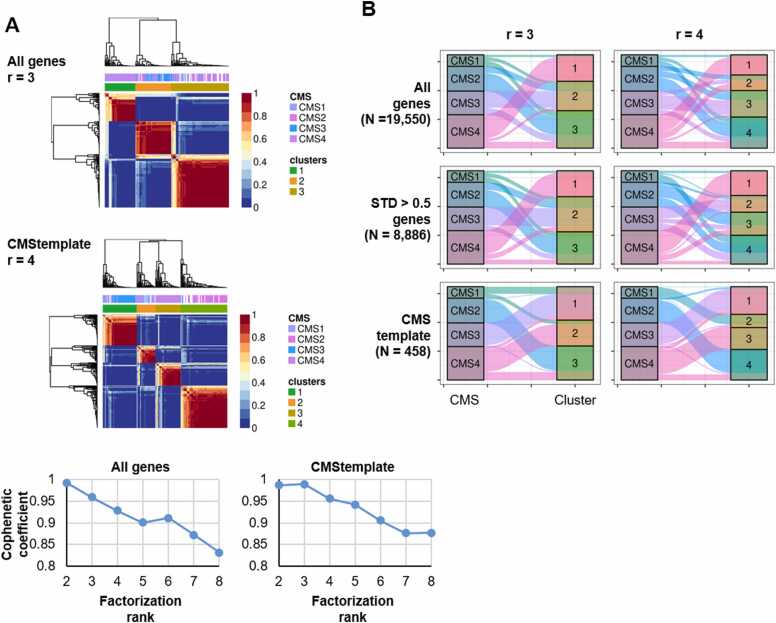
Comparison of CMS classification and NMF-based classification of RNA-Seq data from the CMCBSN cohort. (A) Classification using NMF was exclusively performed on tumor samples (N = 187) within the CMCBSN cohort, presented as a heatmap with factorization rank (*top*). The white color denotes NA values. The top heatmap corresponds to genes expressed in more than 10% of the total samples, with “r” set to “3.” The bottom heatmap represents CMS template genes with “r” set to “4.” The cophenetic coefficient varies with the number of clusters in the NMF clustering for all genes and CMS templates (*bottom*). (B) Sankey plot illustrating the categorization of genes: top, genes expressed in more than 10% of the total samples; middle, genes with a standard deviation of 0.5 or higher among these; and bottom, CMS template genes. The plot on the left corresponds to “r” set to “3,” whereas the one on the right corresponds to “r” set to “4.”

We compared the CMS classification with the NMF-based classification of RNA-Seq data from the CMCBSN cohort, focusing on the transcriptomic profile difference between the Korean and Western populations. The molecular subtyping analysis specifically targeted tumor samples (*N* = 187) within the CMCBSN cohort. This analysis utilized consensus clustering through NMF, summarized by the cophenetic coefficient. Genes were categorized into 3 groups: those expressed in more than 10% of total samples, those with a standard deviation of 0.5 or higher, and CMS template genes. The factorization rank estimation was 3 (Figs. 4A, S5, and S6).

A comparison of the obtained results with CMS subtypes revealed slight differences. At r = 3, CMS2, CMS3, and CMS4 were reasonably well distinguished, whereas CMS1 showed some overlap with CMS3 (Fig. 4B, *left*). When r = 4 was used for the comparison of the obtained results with the 4 CMS groups, the most distinct separation occurred when focusing on CMS template genes (Fig. 4B, *right bottom*). In conclusion, when dividing into 4 groups based on template genes through the CMS subtype classification, the results closely aligned with the CMS subtype outcomes. This consistency suggests that the subtypes identified in our CMCBSN cohort share key molecular features with their Western counterparts, supporting a robust and comparable molecular classification across different populations.

### Epigenetic Exploration of DEGs Between Normal and Tumor Samples and CMS Subtype Genes Showing Disparity

**Fig. 5:**
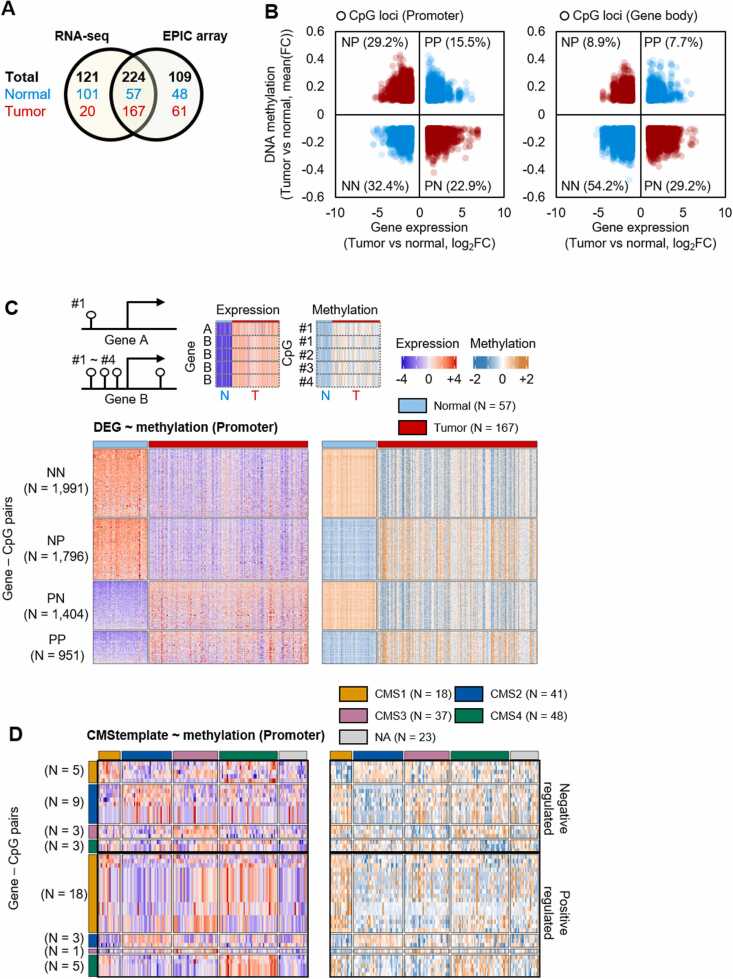
Epigenetic exploration of DEGs between normal and tumor samples and CMS subtype genes showing disparity. (A) The number of CMCSBN samples commonly generated from RNA-Seq and EPIC array. (B) Correlation between log_2_FC of DEGs and FC of DMPs for tumor vs normal samples. Promoter CpG (left), gene body CpG (right). (C) Heatmap illustrating the relationship between gene expression and DNA methylation analyzed in Figure 5B (promoter). The heatmap categories were determined based on the correlation between increased gene expression and DNA methylation in tumor versus normal samples, resulting in positive-positive (PP), negative-positive (NP), positive-negative (PN), and negative-negative (NN) categories. In cases where a single gene had multiple probes, each probe was represented on a separate line in the heatmap to ensure the comprehensive display of all probes (*top*). (D) Heatmap representing the correlation between DMPs based on CMS template genes (promoter). Gene-CpG pairs showing negative or positive correlation with DNA methylation for each CMS subtype were categorized and are presented in the heatmap.

DNA methylation analysis of CRC and CMS subtypes is crucial owing to the pivotal role of DNA methylation in CRC development, influencing the molecular subtype classification, serving as a prognostic indicator, and offering potential therapeutic targets (Cervena et al., 2020). Understanding DNA methylation status aids in predicting CRC treatment outcomes, addressing chemoresistance challenges, and exploring therapeutic interventions such as DNMT inhibitors for patients with CRC, emphasizing the relevance of epigenetic compositions in therapy response variations among individuals. In a previous study, we analyzed the methylome in the CMCBSN cohort (Lee et al., 2023). In the present study, aiming to elucidate the mechanisms of epigenetic regulation concerning DEGs between normal and tumor samples (Fig. 2B) and CMS subtypes (Fig. 3A and Table S7), we performed an analysis of samples for which both RNA-Seq and EPIC array data were simultaneously generated (Fig. 5A, *N* = 224). The gene-CpG pairs were categorized based on the relationship between increased gene expression and DNA methylation in tumor versus normal samples, resulting in positive-positive (PP), negative-positive (NP), positive-negative (PN), and negative-negative (NN) categories (Fig. 5B). In promoters, the results showed that the negative correlation between expression and methylation (PN + NP = 52.1%) was higher than the positive correlation (PP + NN = 47.9%; Fig. 5B, *left*; Fig. 5C). Conversely, in the gene body regions, the negative correlation (PN + NP = 38.1%) between expression and methylation was lower than the positive correlation (PP + NN = 61.9%; Fig. 5B, *right*; Fig. S7A). Additionally, the analysis of CMS template genes revealed genes whose expression had an associated methylation pattern (Figs. 5D and S7B). In conclusion, this analysis revealed differentially methylated genes between DEGs and CMS subtypes. These findings shed light on the intricate interplay between gene expression and DNA methylation, providing a deeper understanding of the epigenetic landscape in the context of cancer and normal tissue variations.

## DISCUSSION

In this study, we analyzed RNA-Seq data from 214 Korean participants (comprising a total of 381 samples, including 169 normal and 212 tumor samples) with CRC to make it available to other researchers. The data included read counts, gene expression matrices, and clinical data. In addition, we used CMScaller to obtain CMS information for each sample. We applied 2 filtering steps based on the mapping ratio and sample mixing, which can be adjusted according to the user's preference. We have provided data before and after filtering for read count and normalized gene expression data.

Our investigation yielded noteworthy findings, particularly demonstrating upregulated IL-17 expression in tumor cells compared with that in their normal counterparts. IL-17, a proinflammatory cytokine, is relevant in the context of not only angiogenesis in CRC (Razi et al., 2019) but also CRC prognosis (Schetter et al., 2009). As substantiated by previous study findings (Qi et al., 2015, Tseng et al., 2014), targeting IL-17 through therapeutic interventions is a potential CRC treatment strategy.

Traditionally, the prognosis of CRC has relied on pathological studies encompassing histological assessments, such as the TNM stage, and evaluations of lymphatic, vascular, or neural invasion. However, the landscape of CRC research has evolved substantially following the publication of the CMS classification in 2015 (Guinney et al., 2015). Subsequent studies have explored tumor prognosis according to the CMS classification. CMS1 has a poorer prognosis than other CMS subtypes, as previously reported (Ten Hoorn et al., 2022). Our CMCBSN cohort did not show distinct survival patterns in the survival analysis. This lack of a pattern may be attributed to the higher proportion of patients in the survival group than in the deceased group (89.95% vs 10.05%, respectively). In addition, the absence of reported mortality within the CMS1 subgroup further contributes to the absence of clear survival patterns. When independently dividing subgroups, the estimated rank “r” was determined to be 3; however, this result might have been influenced by the small size of the CMCBSN tumor samples (*N* = 187) and the relatively low representation of the CMS1 subtype. When the analysis was specifically conducted using CMS template genes with “r” set to 4, the subtypes closely aligned with the CMS classification. Consequently, it can be inferred that the CMCBSN cohort exhibits substantial similarity to Western cohorts, with the primary discrepancy likely attributed to the smaller sample size and the relative scarcity of the CMS1 subtype samples within the CMCBSN tumor samples. To conduct more precise analyses, data collection from larger cohorts is necessary.

In conclusion, we conducted a transcriptome analysis of a cohort of 214 Korean participants. To underscore the utility of our dataset, we provided data for each data-processing stage. Our findings align with previously established results from CRC transcriptome analyses, confirming the consistency and reliability of our data.

## Author Contributions

Young-Joon Kim, Jin Ok Yang, and Kil-yong Lee conceived and designed the study. Jaeim Lee, Hoang Bao Khanh Chu, Seong-Taek Oh, Sung-Bum Kang, Sejoon Lee, Duck-Woo Kim, Heung-Kwon Oh, Jisun Kang, Jin-Young Lee, Sheehyun Cho, Hyeran Shim, Hong Seok Lee, and Kil-yong Lee collected the data. Jaeim Lee, Jong-Hwan Kim, Hoang Bao Khanh Chu, Jisu Kim, Jisun Kang, and Kil-yong Lee contributed data or analysis tools. Jong-Hwan Kim, Ji-Hwan Park, Jisu Kim, and Kil-yong Lee performed the analysis. Jaeim Lee, Ki.J.H., Hoang Bao Khanh Chu, and Kil-yong Lee wrote the manuscript.

## Declaration of Competing Interests

The authors declare that they have no known competing financial interests or personal relationships that could have appeared to influence the work reported in this paper.

## Data Availability

The RNA-Seq data have been deposited in the Korean Nucleotide Archive under the accession number KAP230608 (https://www.kobic.re.kr/kona/search_bioproject?bioproject_id=KAP230608#none). Clinical data, preprocessed gene quantification (RNA-Seq by Expectation-Maximization), normalized gene expression data (log_2_CPM), and downstream analysis results have been deposited in Zenodo (https://doi.org/10.5281/zenodo.8333650). Further information and requests for experimental resources should be directed to and will be fulfilled by the corresponding authors, Kil-yong Lee (cyboryee@hanmail.net), Jin Ok Yang (joy@kribb.re.kr), and Young-Joon Kim (yjkim@yonsei.ac.kr).

## Appendix A Supplementary material

Supplementary Table 1 Clinical characteristics of the patients in the St. Mary's Hospital and Bundang Seoul National University Hospital cohort.

.

Supplementary Table 2 Quality metrics for RNA-Seq data of the St. Mary's Hospital and Bundang Seoul National University Hospital cohort.

.

Supplementary Table 3 Quality metrics for RNA-Seq data of the St. Mary's Hospital and Bundang Seoul National University Hospital cohort.

.

Supplementary Table 4 Gene set enrichment analysis results comparing tumor and normal samples.

.

Supplementary Table 5 Statistical validation of clinical information between the Tumor-weak and remaining tumor groups.

.

Supplementary Table 6 Differentially expressed genes among Consensus Molecular Subtype groups.

.

Supplementary Table 7 Differentially methylated genes derived from DEGs and their association with CMS subtypes in both promoter and gene body regions.

.

Supplementary Fig. 1 (A) Phred quality scores for all reads in this study. (B) Density plot of uniquely mapped reads in the St. Mary's Hospital and Bundang Seoul National University Hospital cohort.

.

Supplementary Fig. 2 (A) Heatmap showing differentially expressed genes between tumor and normal tissues using k-means clustering. (B) Multidimensional scaling plot comparing the St. Mary's Hospital and Bundang Seoul National University Hospital cohort before and after filtering. (C) Gene set enrichment analysis of Gene Expression Omnibus signatures (RNA-sequencing). (D) Identification of Tumor-weak samples through k-means clustering (k = 10). (E) Statistical validation of clinical information between the Tumor-weak and remaining tumor groups.

.

Supplementary Fig. 3 (A) Survival analysis of Consensus Molecular Subtype (CMS) groups. (B) Survival analysis was performed between the CMS1+4 and CMS2+3 groups. (C) Differentially expressed gene analysis between the CMS and non-CMS groups.

.

Supplementary Fig. 4 (A) Results of survival analysis conducted with clinical information using a forest plot.

.

Supplementary Fig. 5 Quality measures computed from 500 runs for the estimation of each “r” value. (A) Genes expressed in more than 10% of the total samples. (B) Genes with a standard deviation of 0.5 or higher among these (C) CMS template genes.

.

Supplementary Fig. 6 Consensus matrices computed from 500 runs for each “r” value. (A) Genes expressed in more than 10% of the total samples. (B) Genes with a standard deviation of 0.5 or higher among these (C) CMS template genes.

.

Supplementary Fig. 7 (A) Heatmap illustrating the relationship between gene expression and DNA methylation analyzed in Fig. 5B (gene body). The heatmap categories were determined based on the correlation between increased gene expression and DNA methylation in tumor versus normal samples, resulting in positive–positive (PP), negative–positive (NP), positive–negative (PN), and negative–negative (NN) categories. (B) Heatmap representing the correlation between DMPs based on CMS template genes (gene body). Gene–CpG pairs showing negative or positive correlation with DNA methylation for each CMS subtype were categorized, and they are presented in the heatmap.
