# Supplementary figures and images for: Comprehensive RNA-sequencing analysis of colorectal cancer in a Korean cohort
Source: Mol Cells. 2024 Feb 23;47(3):100033. doi: 10.1016/j.mocell.2024.100033 (PMC11004400; doi:10.1016/j.mocell.2024.100033)

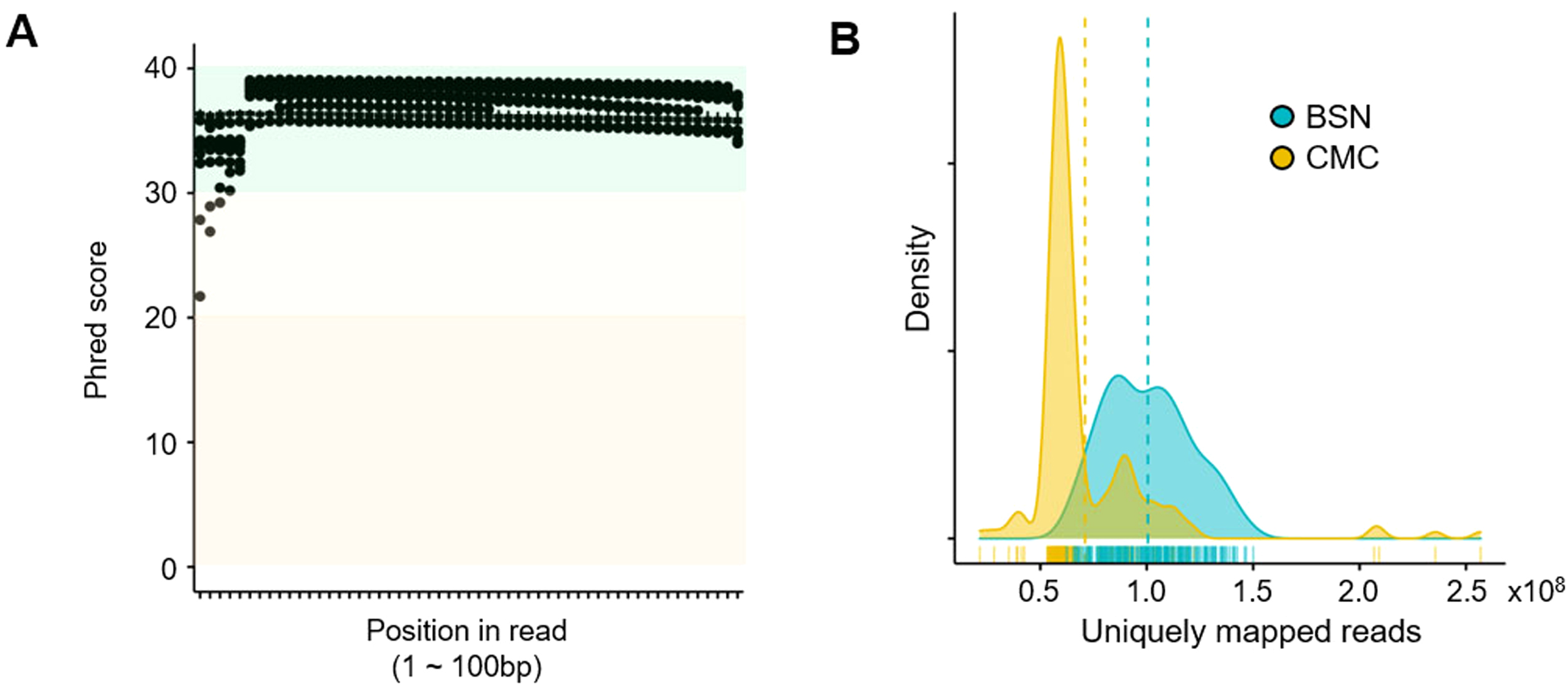

Supplement: Supplementary file 8 — Supplementary Fig. 1 (A) Phred quality scores for all reads in this study. (B) Density plot of uniquely mapped reads in the St. Mary's Hospital and Bundang Seoul National University Hospital cohort. [file mmc8.jpg]

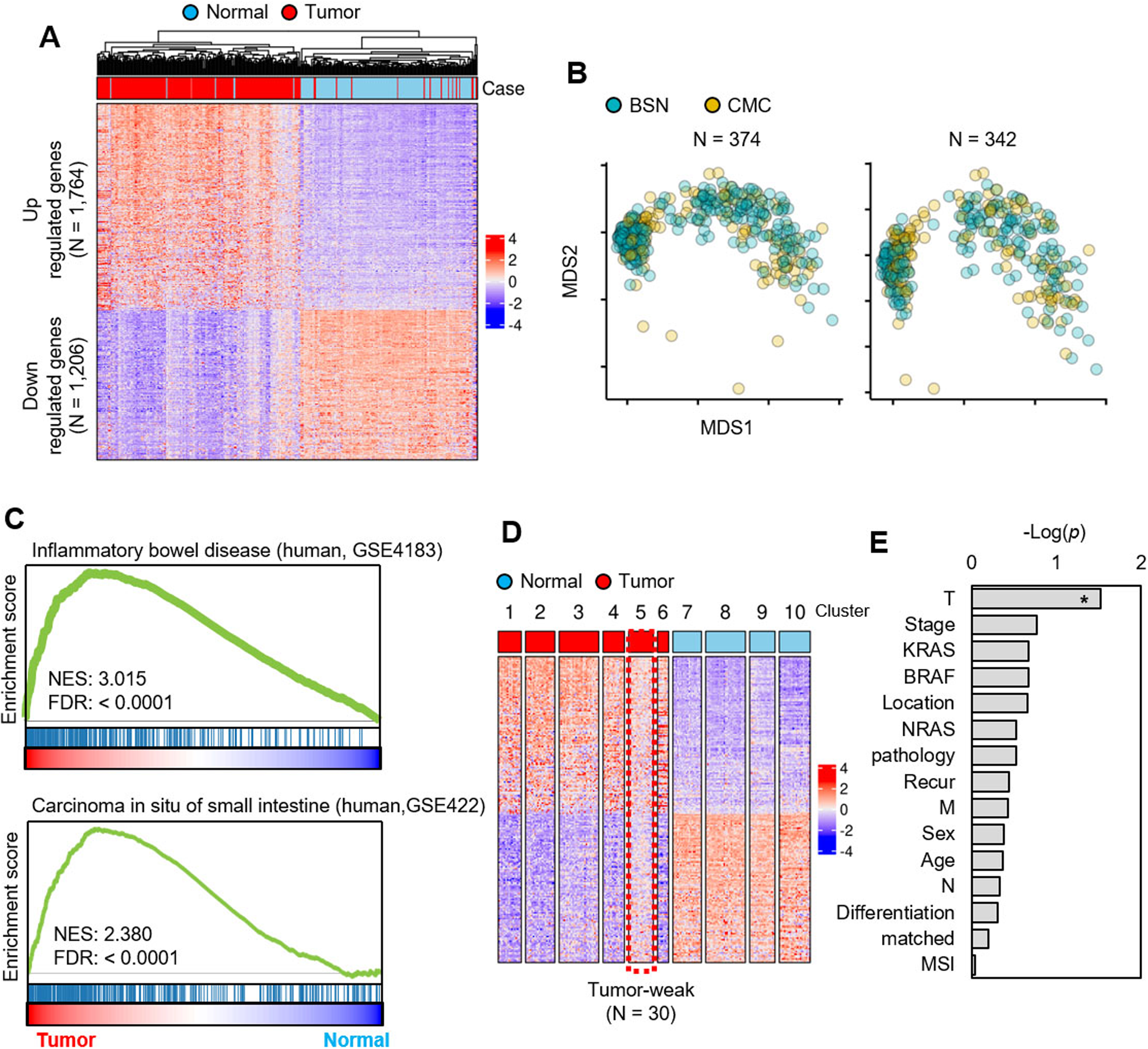

Supplement: Supplementary file 9 — Supplementary Fig. 2 (A) Heatmap showing differentially expressed genes between tumor and normal tissues using k-means clustering. (B) Multidimensional scaling plot comparing the St. Mary's Hospital and Bundang Seoul National University Hospital cohort before and after filtering. (C) Gene set enrichment analysis of Gene Expression Omnibus signatures (RNA-sequencing). (D) Identification of Tumor-weak samples through k-means clustering (k = 10). (E) Statistical validation of clinical information between the Tumor-weak and remaining tumor groups. [file mmc9.jpg]

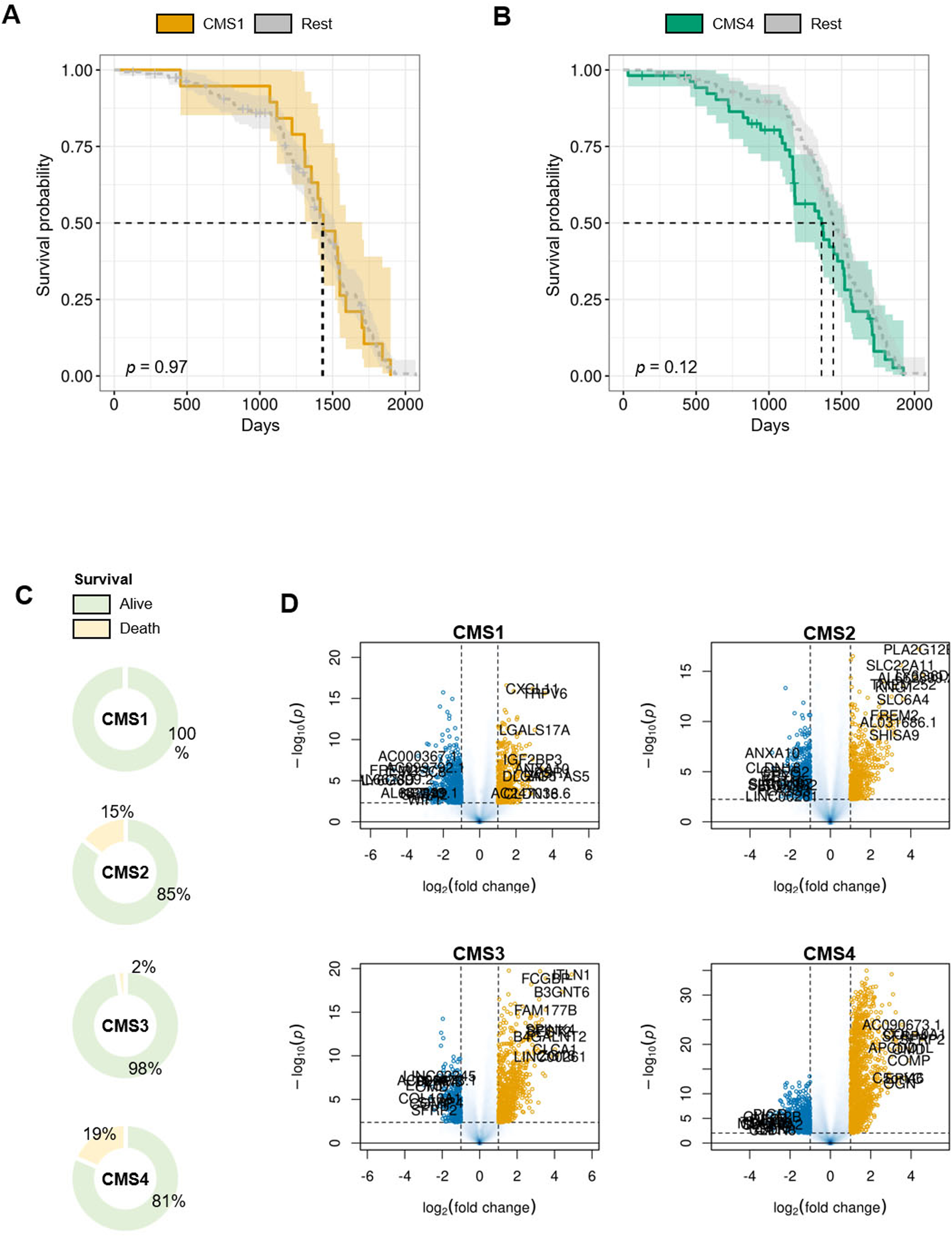

Supplement: Supplementary file 10 — Supplementary Fig. 3 (A) Survival analysis of Consensus Molecular Subtype (CMS) groups. (B) Survival analysis was performed between the CMS1+4 and CMS2+3 groups. (C) Differentially expressed gene analysis between the CMS and non-CMS groups. [file mmc10.jpg]

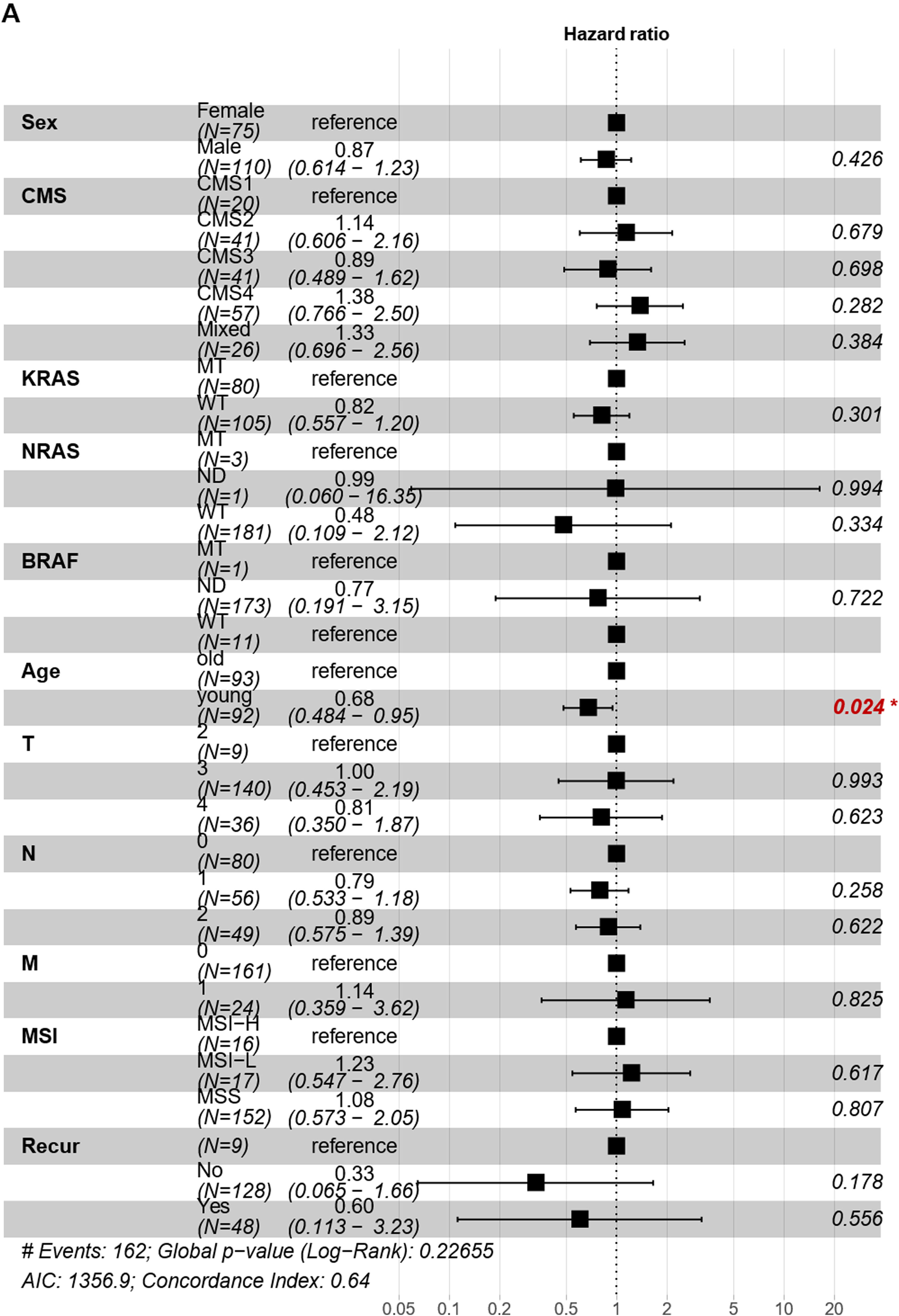

Supplement: Supplementary file 11 — Supplementary Fig. 4 (A) Results of survival analysis conducted with clinical information using a forest plot. [file mmc11.jpg]

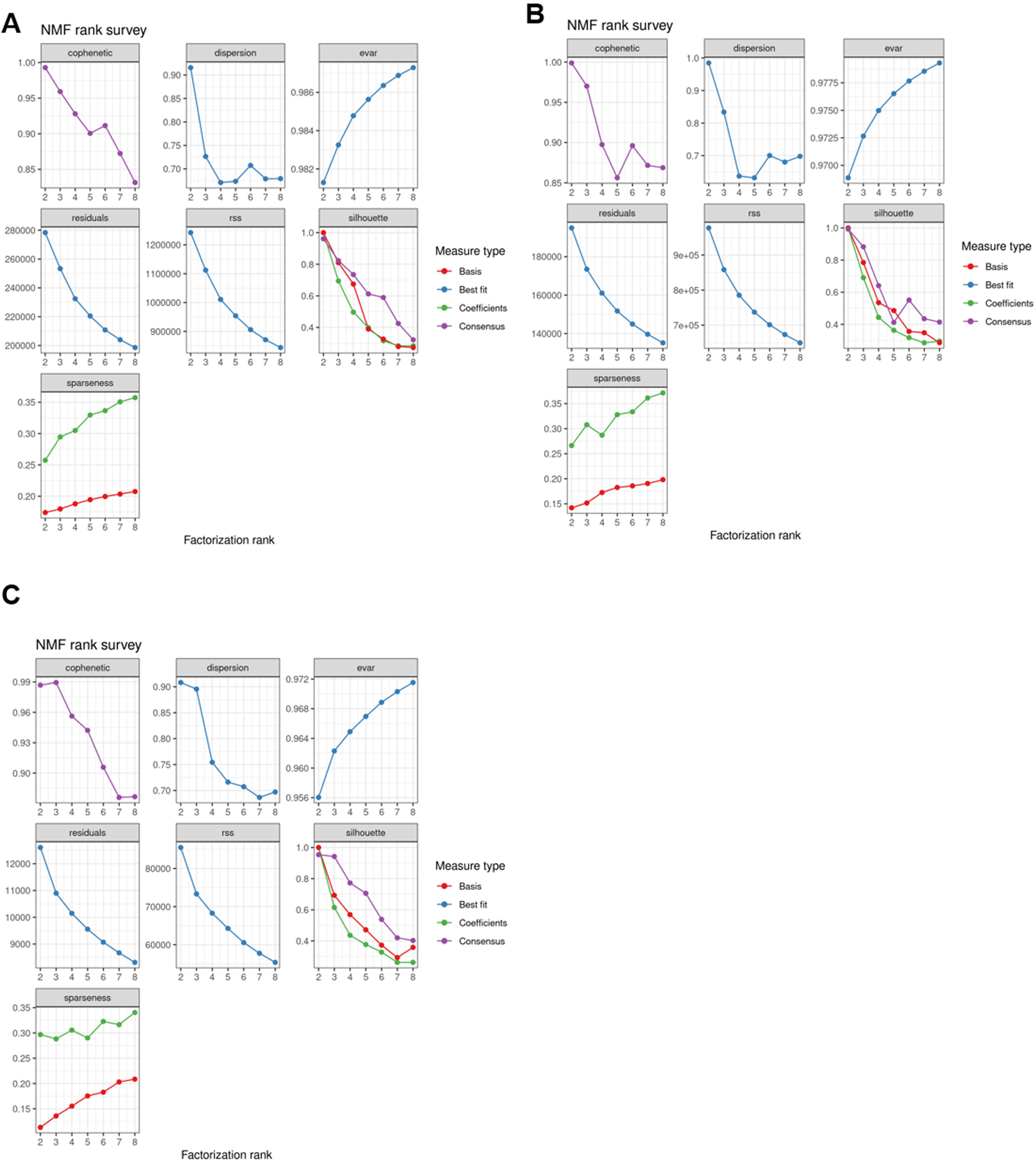

Supplement: Supplementary file 12 — Supplementary Fig. 5 Quality measures computed from 500 runs for the estimation of each “r” value. (A) Genes expressed in more than 10% of the total samples. (B) Genes with a standard deviation of 0.5 or higher among these (C) CMS template genes. [file mmc12.jpg]

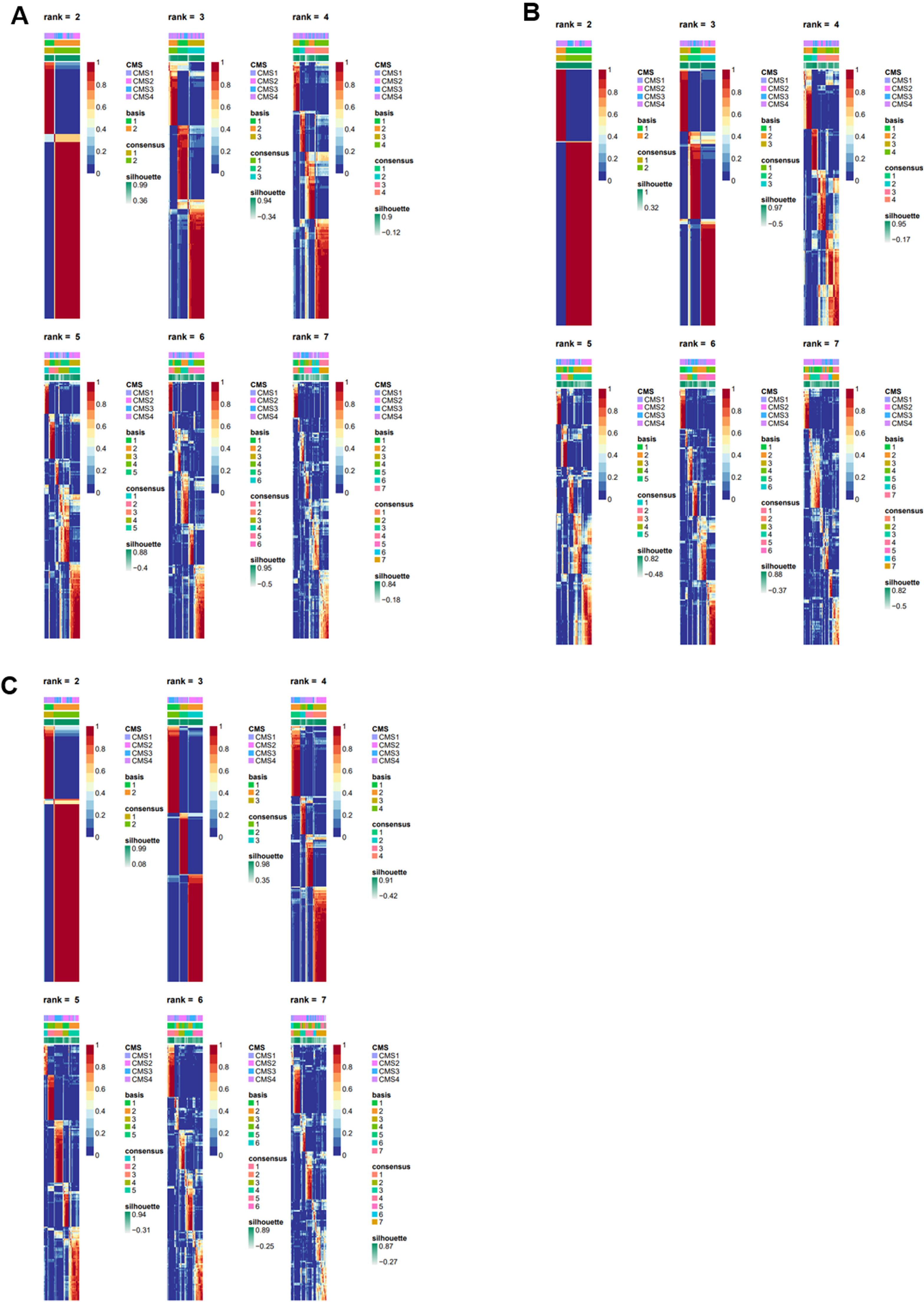

Supplement: Supplementary file 13 — Supplementary Fig. 6 Consensus matrices computed from 500 runs for each “r” value. (A) Genes expressed in more than 10% of the total samples. (B) Genes with a standard deviation of 0.5 or higher among these (C) CMS template genes. [file mmc13.jpg]

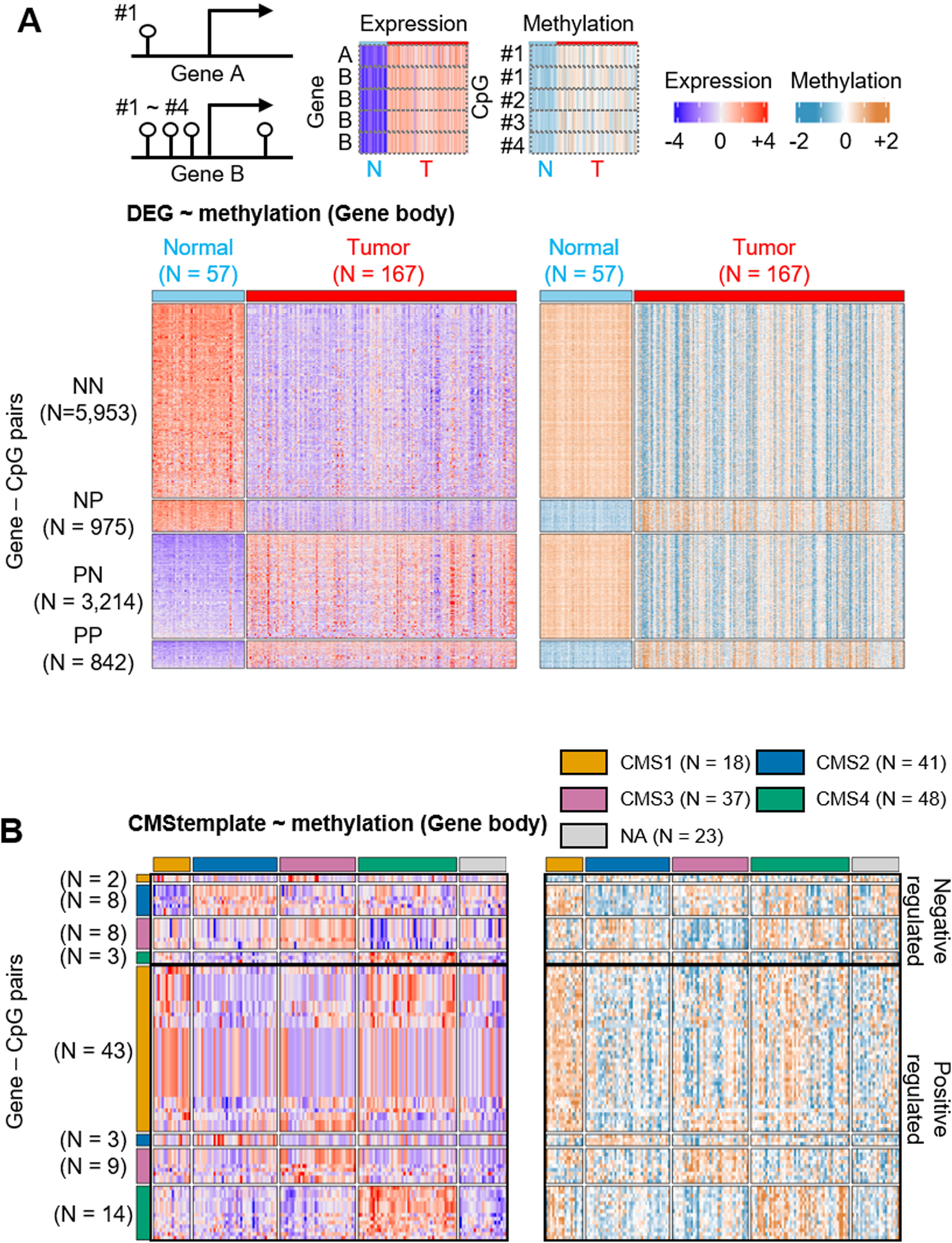

Supplement: Supplementary file 14 — Supplementary Fig. 7 (A) Heatmap illustrating the relationship between gene expression and DNA methylation analyzed in Fig. 5B (gene body). The heatmap categories were determined based on the correlation between increased gene expression and DNA methylation in tumor versus normal samples, resulting in positive–positive (PP), negative–positive (NP), positive–negative (PN), and negative–negative (NN) categories. (B) Heatmap representing the correlation between DMPs based on CMS template genes (gene body). Gene–CpG pairs showing negative or positive correlation with DNA methylation for each CMS subtype were categorized, and they are presented in the heatmap. [file mmc14.jpg]
